# X-ray microtomography provides first data about the feeding behaviour of an endangered lizard, the Montserrat galliwasp (*Diploglossus montisserrati*)

**DOI:** 10.1098/rsos.150461

**Published:** 2015-12-23

**Authors:** C. Bochaton, R. Boistel, L. Charles

**Affiliations:** 1 Laboratoire Archéozoologie et Archéobotanique: Sociétés, Pratiques et Environnements, UMR 7209 – CNRS, MNHN, Muséum national d'Histoire naturelle, Sorbonne Universités, 55 rue Buffon, CP 56, Paris 75005, France; 2 Institut de Systématique, Évolution, Biodiversité, ISYEB – UMR 7205 – CNRS, MNHN, UPMC, EPHE, Muséum national d'Histoire naturelle, Sorbonne Universités, 57 rue Cuvier, CP 30, Paris 75005, France; 3 Institut de Paléoprimatologie, Paléontologie Humaine: Evolution et Paléoenvironnements, UMR 7262–CNRS, Université de Poitiers, UFR SFA, Bât. B35, 6 rue Michel Brunet, TSA 51106, Poitiers 86073, France; 4 Muséum d'Histoire naturelle de Bordeaux, 5 Place Bardineau, Bordeaux 33000, France

**Keywords:** Anguidae, durophagy, insular fauna, predation, West Indies

## Abstract

Reporting the diet of recently extinct or very rare taxa, only known by a few museum specimens, is challenging. This study uses X-ray microtomography, a non-destructive investigation method, to obtain the first data about feeding behaviours in the Montserrat galliwasp (*Diploglossus montisserrati*) by scanning one of the two specimens known to date. The scans revealed the occurrence of shell fragments of a freshwater snail (*Omalonyx matheroni*) in the digestive tract of the specimen. This data combined with morphological evidence shows the occurrence of a durophagous feeding habit and a possible tendency of association with freshwater environments. This information could be crucial to save this critically endangered lizard endemic on Montserrat island.

## 1. Introduction

The feeding behaviours and diet are part of the main characteristics that typify animals. They provide information about the trophic relationships between organisms in addition to their lifestyle, habitat use and life history. This is also true for squamates, and several techniques exist to gain an understanding of their diet. For living animals, feeding behaviour can be observed in the field [1] or in captivity [2], when it cannot be directly observed, by using stomach flushing [3] or by collecting faecal samples [4]. With dead specimens, a dissection can be performed to observe the stomach contents of fresh or alcohol-preserved specimens [5]. Finally, isotopic analysis [6] and inferences based on dental morphology [7] can be done on living, dead and fossil specimens. However, which is the method to apply in the case of unique or rare museum specimens? Techniques requiring tissue sampling (isotopic analysis) or that induce damage to the specimen (dissection) should be avoided. Moreover, inferences based on anatomical data do not provide accurate information. As a consequence, obtaining data concerning the diet of extinct or nearly extinct species can be difficult even if museum specimens exist. Here, we tested the use of X-ray radiography and X-ray microtomography (XMT) to investigate stomach contents and tooth morphology of a rare museum specimen, the type specimen of the Montserrat endemic galliwasp, *Diploglossus montisserrati* Underwood, 1964. Data concerning this lizard are very rare, and since its description by Underwood in 1964 [8], it has been seen only 12 times in an area of only 1.5 ha [9]. Observers reported that the animal was terrestrial, most probably nocturnal and often found in the vicinity of streams [9,10]. Concerning its diet, Underwood [8] suggested that this galliwasp could possibly feed on young crayfish. However, this suggestion was never verified. Data concerning the ecology of this animal until now have been limited to the few observations made on living galliwasps observed roaming on the ground of moist broadleaf forest. Our results provide the first direct observations on the diet of this, according to the IUCN, critically endangered lizard [11] and could provide useful information to help to preserve this animal [10].

## 2. Material and methods

The scan and the scanned specimen are stored at the Museum of Comparative Zoology (Harvard, MA, USA) (MCZ R-76924). The specimen is the type and one of the two existing specimens of *D. montisserrati*. The second specimen was collected in 2005 and is stored on Montserrat Island (M. Morton 2013, personal communication) but was not available to us. The type specimen we studied was described by Underwood in 1964 and collected by J. Kingsley probably a few years before at Woodland Spring in Montserrat [8]. However, no precise location or collection date was mentioned. This specimen lacks its left lower jaw, which was removed, illustrated and described by Underwood [8]. The snout–vent length of this specimen is 180 mm, a size similar to *D. montisserrati* specimens observed in the wild.

XMT was performed according to the protocols reported in the literature [12–14]. We used a Viscom X8050-16 μCT scanner at the Centre for Microtomography of the University of Poitiers (France). Scans were performed at 100 kV and 34/32 mA. The geometry was set to obtain an 84.2–62.9 μm voxel size in the reconstructed three-dimensional images. The reconstruction was performed using the software ImageJ (http://imagej.nih.gov) [15] and the FDK algorithms of DigiCT v. 2.4.3 (Digisens with plug-in SnapCT, acceleration in GPU). The dataset consists of 2160 projections (720×3) taken over 360° for the whole body and 900 projections for the head of the specimen. Direct volume rendering was used to visualize the sub-set of selected voxels of the prey, the skeleton and the jaw in AVIZO v. 7.1 and 6.1 (VSG, SAS, Merignac, France, http://www.vsg3d.com), after having used ImageJ to mask the anatomical structures we were not interested in.

## 3. Results

### 3.1 Tooth morphology

**Figure 1.**
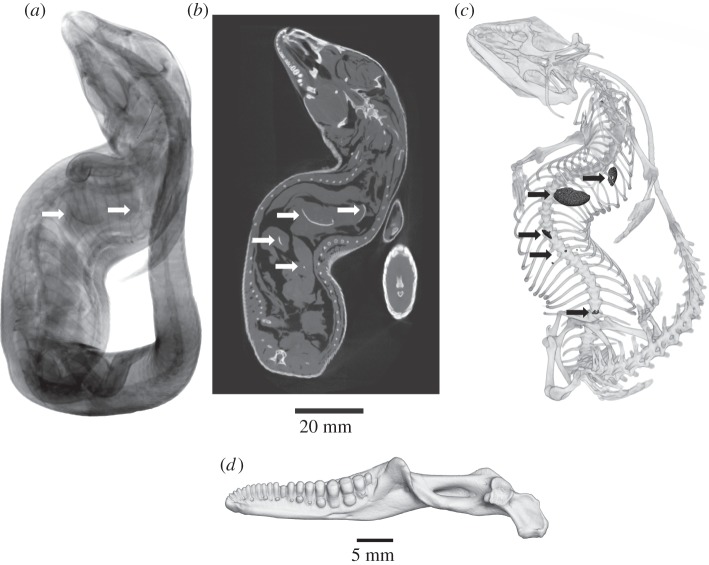
(*a*) X-ray radiography, (*b*) microtomogram and (*c*) direct volume rendering (ventral view) obtained by XMT of *D. montisserrati* (Museum of Comparative Zoology, Harvard University R-76924). The shell remains are barely visible on the radiograph (*a*—white arrow) but well visible on the microtomogram (*c*—white arrows). In volume rendering (*c*) all elements other than bones and stomach content were removed (with the exception of the extremities of the limbs). Shell fragments (in black) are clearly visible in the visceral cavity of the specimen. (*d*) Direct volume rendering of the right mandible presenting molariform teeth (lingual view).

The tooth morphology is similar to what was described by Underwood [8]; the 10 most anterior dental positions contain pointed teeth and the teeth in the five anterior-most positions are slightly posteriorly curved. The more posterior tooth positions bear teeth that tend to become wider in lateral view and the four last teeth are wide and globular molariform teeth (figure 1*d* and electronic supplementary material). We observed the same global organization in the maxilla. Molariform teeth observed on *D. montisserrati* were also reported to appear across the ontogeny in the giant greater Antillean galliwasps *Diploglossus crusculus* (=*Celestus crusculus* Garman, 1887) and *Diploglossus warreni* (=*Celestus warreni* Schwartz, 1970) [16]. The diet of *C. crusculus* remains mainly unknown but *D. warreni* is an opportunistic forager consuming invertebrates and small vertebrates [17].

### 3.2 Stomach contents

Radiography of the specimen showed the occurrence of two large objects in the stomach, but their precise forms could not be distinguished (figure 1*a*). Additional investigations using tomograms and three-dimensional visualizations showed that, in addition to the objects observed with radiography, several objects presenting a similar density could be observed in the digestive tract of the specimens (figure 1). Most of them could not be identified but the larger two observed in the stomach appear to be shell remains, one of which being nearly intact. By deduction, we believe that the other smaller objects observed in the intestine were smaller, broken and partially digested fragments of shells. Digestion of shell is unsurprising for durophagous lizards and was previously recorded for *Tupinambis* [18] and chameleons [19].

**Figure 2.**
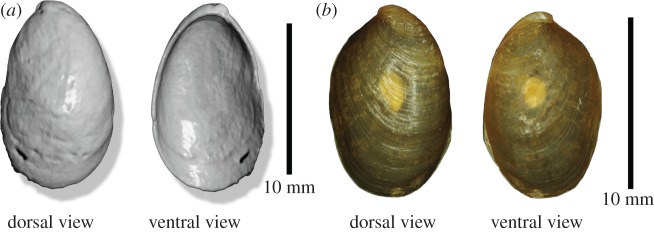
Comparison of the most complete shell fragment observed in the stomach of *D. montisserrati* (*a*) (volume rendering obtained from XMT) with an *O. matheroni* shell from Martinique (*b*) (Muséum d'Histoire naturelle de Bordeaux MHNBx 2015.9.1).

The most complete shell measures 12 mm in length and was identified using comparative specimens stored in the natural history museum of Bordeaux (France) (figure 2). It presents a very short spire and a large first whorl that suggest an attribution to the genus *Omalonyx* d'Orbigny, 1837. This genus is widely distributed in South America but only one species currently occurs in the Antilles, *Omalonyx matheroni* (Potiez & Michaud, 1835) [20]. This species is considered to have been introduced in Guadeloupe, Martinique and Dominica [21]. Historically, the occurrence of this genus in Montserrat has been reported since 1894 [22] and so was already present on the island when the MCZ *D. montisserrati* specimen was collected. Consequently, we propose an attribution of this shell to the species *O. matheroni*.

## 4. Discussion

The observation of a shell of *O. matheroni* in the digestive tract of *D. montisserrati* constitutes the first direct observation of the diet in this IUCN critically endangered endemic galliwasp.

The identification of this prey provides the only information about the feeding habits of *D. montisserrati*. Indeed, the members of the genus *Omalonyx* are amphibious land snails that have a distribution that is strongly limited to the vicinity of freshwater. They are observed on submerged vegetation, on the shores of lagoons, and also in moist bushes near water reservoirs [23,24]. Consequently, our observation may indicate that *D. montisserrati* might occasionally hunt near freshwater. Since *Omalonyx* does not live underwater, our observations do not indicate that *D. montisserrati* is an aquatic feeder. However, considering that *Omalonyx* was recently introduced in Montserrat, *D. montisserrati* could also feed on other freshwater species or species associated with freshwater as previously suggested by Underwood [8]. Other soft-bodied prey indiscernible using XMT may also occur in the digestive tract of the specimen and could possibly be tracked by phase-contrast imaging using synchrotron XMT. Unfortunately, magnetic resonance imaging cannot be used for our specimen because of the occurrence of metallic pins in its body [14].

The dental morphology of *D. montisserrati*, with large and globular posterior teeth on the dentary and our observation of shell fragments inside its digestive tract, confirms the association between molariform teeth and hard food, as has been observed for other lizards [16,25–27]. In West Indian Diploglossinae, such teeth were only observed in large species of which at least one (*D. warreni*) is an opportunistic forager consuming a broad range of soft (insects) and hard prey (vertebrates) but no molluscs or crustaceans [17]. In contrast, two other galliwasps, *Diploglossus millepunctatus* [28] and *Diploglossus monotropis* in captivity [10], are known to eat molluscs or crustaceans but both appear to lack molariform teeth, as was also observe in juvenile *Chamaeleolis* lizards [29]. Obviously, quantitative data concerning the diet and tooth morphology of West Indian Diploglossinae, as well as for many other lizards, are still too scant to clearly assess how these two traits are linked. However, overall, these data suggest that the occurrence of molariform teeth in insular lizards might be an adaptation allowing a broader range of prey to be taken in a limited environment [16].

To conclude, although our observation represents an isolated case and is too limited to provide detailed information on the ecology of *D. montisserrati*, it provides the only available dietary data of this endangered galliwasp species. Our results show that the particular habitat type near freshwater may be targeted in order to observe *D. montisserrati* specimens in the wild, which could allow direct observations of its feeding and foraging behaviour. This is important because only a better understanding of the ecology of this unique animal can help improve action plans and may help prevent its possible extinction in the very near future.

## Data accessibility

Our scan of the type specimen of *D. montisserrati* is stored by the Museum of Comparative Zoology (Harvard, USA) and can be requested from this institution.
